# Distinctive physiology of polyphosphate-accumulating *Beggiatoa* suggests an important role in benthic phosphorus cycling

**DOI:** 10.1128/aem.02330-24

**Published:** 2025-04-30

**Authors:** Nadezhda Iakovchuk, Jenny Fabian, Olaf Dellwig, Christiane Hassenrück, Heide N. Schulz-Vogt

**Affiliations:** 1 Department of Biological Oceanography, Leibniz Institute for Baltic Sea Research Warnemünde 28389, Rostock, Germany; 2 Faculty of Mathematics and Natural Sciences, University of Rostock 98914 , Rostock, Germany; 3 Department of Marine Geology, Leibniz Institute for Baltic Sea Research Warnemünde 28389, Rostock, Germany; University of Delaware, Lewes, Delaware, USA

**Keywords:** marine sediments, sulfur bacteria, phosphate starvation, phosphate uptake, polyphosphate, coastal ocean

## Abstract

Filamentous sulfide-oxidizing *Beggiatoa* spp., which occasionally form extensive white microbial mats, are widespread in marine coastal environments and can achieve significant biomass because of their large size. Their ability to store phosphates in the polymerized form of polyphosphates makes them potential key players in altering the phosphorus (P) cycle at the sediment-water interface. This study examined phosphate uptake and polyphosphate formation in a P-starved culture of *Beggiatoa* sp. 35Flor strain. Remarkably, even after sustained P starvation over five generations, the mat establishment rate of the examined culture was 46%, demonstrating considerable plasticity in response to different levels of phosphate availability. Under these P-depleted conditions, at least 17% of filaments still contained polyphosphates, highlighting their critical role in their metabolism. Upon reintroduction of phosphate to starved cultures, an extremely rapid phosphate uptake was observed within the first 10 min, with rates reaching up to 12.4 mmol phosphate g^−1^ protein h^−1^, which is significantly higher than values previously reported in the literature for similar-sized organisms. The high phosphate uptake capacity of *Beggiatoa* spp., estimated at 0.6–6 mmol m^−2^ d^−1^ for typical densities of filaments in coastal sediments, suggests that under certain environmental conditions, these bacteria could act as a P sink and thus play an important role in benthic P cycling.

**IMPORTANCE:**

Sulfide-oxidizing bacteria of the genus *Beggiatoa* occur ubiquitously in marine coastal sediments and have a large potential to influence phosphate fluxes at the sediment-water interface, owing to their ability to accumulate polyphosphate and their large size. However, the extent to which these bacteria can contribute to phosphorus (P) sequestration or release remains poorly assessed. The importance of this study lies in demonstrating the remarkable flexibility in the adaptation of the strain *Beggiatoa* sp. 35Flor to varying P availability, including prolonged P starvation and its capacity to rapidly uptake and store available phosphate in the form of polyphosphate. When considered on a global scale, these physiological traits could form the basis for *Beggiatoa*'s role in moderating P fluxes.

## INTRODUCTION

Phosphorus (P) is a vital micronutrient essential for the composition of key biomolecules that govern cellular processes, such as replication, energy transfer, and structural integrity; thus, it is often a limiting factor for living organisms (1). In aquatic environments, P availability is closely linked to iron (Fe) because of the affinity of phosphate for solid Fe oxyhydroxides and is primarily driven by changes in redox conditions (2). Under oxic conditions, phosphate adsorbs onto Fe oxyhydroxides that co-precipitate in the uppermost sediment layer, making phosphate unavailable to most organisms. Conversely, under sulfidic conditions, these Fe oxyhydroxides are reduced, thereby releasing bound phosphate back into the porewater. In addition to chemical phosphate binding and release, it is becoming increasingly clear that biological processes, such as bacterial polyphosphate (polyP) accumulation, have a significant influence on P availability in the aquatic systems (3, 4). Although many studies have addressed the importance of sediment microbial communities on P dynamics, the significance of polyP-accumulating bacteria remains underestimated (5–7).

Polyphosphate is a linear molecule composed of multiple orthophosphate units connected by energy-rich phosphoanhydride bonds (8). Prokaryotic organisms synthesize and store polyP in the form of granules, which may be decomposed into orthophosphates or directly utilized for multiple functions (9). Polyphosphates have been recognized as intracellular energy buffers and metabolic regulators; consequently, they have been extensively studied in various prokaryotic organisms (10–13).

With respect to phosphate availability, three distinguishable conditions that stimulate polyP synthesis in microorganisms have been defined in the literature. Under excess phosphate, luxury uptake occurs through the accumulation of polyP beyond its immediate metabolic needs for future use when phosphate becomes limited (14, 15). Conversely, overplus uptake was described when phosphate-deprived cells were re-exposed to phosphate and rapidly accumulated polyP at levels greater than those seen in luxury uptake (16). Additionally, a process termed deficiency or starvation response has been recognized for the ability of microorganisms to form polyP, even under low phosphate conditions (17, 18), although the physiological benefits of this process are not yet fully understood (19).

Notably, large sulfur bacteria accumulate polyP extensively owing to their size and thus can significantly affect P fluxes in marine sediments, thereby impacting local and global biogeochemical cycles. Such cases have been described for the Namibian shelf, where the species *Thiomargarita namibiensis* was associated with the formation of phosphorites (20, 21). Among others, the filamentous large sulfur bacteria *Beggiatoa* sp. 35Flor strain was described to cause significant phosphate release under high sulfide fluxes in the absence of oxygen in laboratory experiments (22), which was later observed in some marine environments (23). Although a systematic estimation of the density of *Beggiatoa* spp. filaments is available only for restricted locations (24–26), their presence has been reported globally in coastal marine sediments (27–33). Considering their ubiquitous nature, the role of these bacteria in the P cycle is highly under-studied, especially in environments where they do not form a distinct white mat on the sediment surface but are still present in significant numbers within the sediment. Investigating the response of *Beggiatoa* spp. to different levels of phosphate availability remains a critical gap in understanding their potential role in benthic P cycling.

The main objective of this study was to investigate the response of P-starved *Beggiatoa* spp. to excess phosphate in order to evaluate the capacity of bacterial mats to moderate sudden nutrient pulses. We specifically addressed two main questions: (i) How does a lack of phosphate influence the propagation of a *Beggiatoa* spp. culture across successive generations? and (ii) What are the dynamics of overplus phosphate uptake and subsequent luxury polyP formation after P reintroduction? To answer these questions, we conducted laboratory experiments in which we subjected the marine strain *Beggiatoa* sp. 35Flor to P starvation for several generations. Subsequently, we introduced an excess of phosphate into the bacterial mat and monitored both phosphate uptake and polyP accumulation. To the best of our knowledge, this is the first attempt to study P starvation and the subsequent polyP accumulation during overplus phosphate uptake in *Beggiatoa* species.

## MATERIALS AND METHODS

### Culture and cultivation

The experiment was conducted on the filamentous marine sulfide-oxidizing *Beggiatoa* sp. 35Flor strain brought into culture in 2002. It was originally isolated from a single filament of an enriched microbial community from black band disease in scleractinian corals along the coast of Florida (34). The chemolithoautotrophic *Beggiatoa* sp. strain 35Flor has a filament thickness of 6 µm and is characterized by distinct polyP and sulfur inclusions (22). It is accompanied by the heterotrophic and metabolically versatile bacterium *Pseudovibrio* sp. strain FO-BEG1, which is present in low biomass and is required for the growth of *Beggiatoa* sp. 35Flor under culture conditions (35).

The co-culture is maintained in a two-layer mineral semi-liquid agar medium with an opposed gradient of sulfide and oxygen as described previously (36). It was used as the initial inoculum for growing P-depleted cultures. P-depleted cultures were acquired by cultivating bacteria in modified media without any additional P sources as follows: agar was washed three times with 18.2 MΩ cm water to minimize P contamination, and the final agar concentration of the top layer was lowered to 0.2%. The bottom agar layer was supplied with a sulfide concentration of 4 mmol L^−1^. Because of the high phosphate concentration (approximately 180 µmol L^−1^) in the initial stock cultures and the internal storage of polyP in the filaments, it took five generations to acquire cultures in which most of the filaments were free of detectable polyP inclusions, as confirmed by microscopic observations (see below: Polyphosphate visualization).

**Fig 1:**
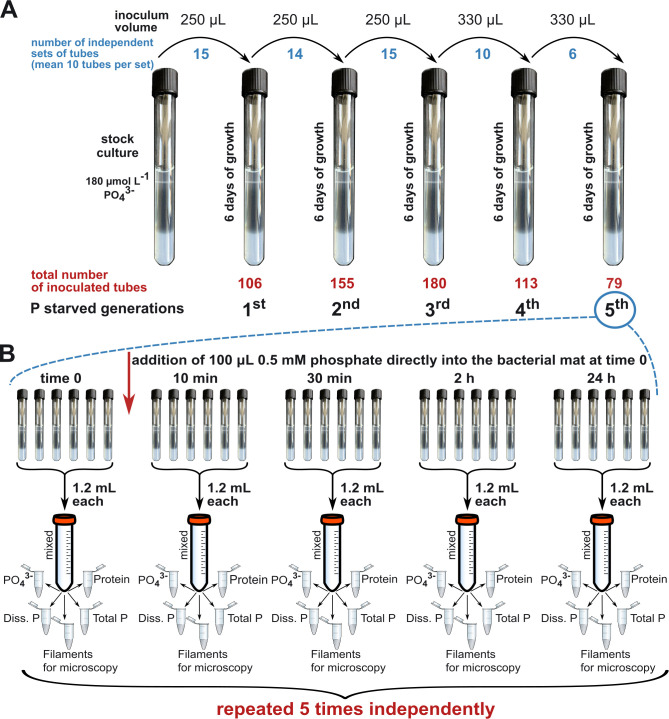
Schematic of the experimental design used in this study. (**A**) Phosphorus starvation over five generations. Each generation was cultivated for 6 days, with a specified amount of culture transferred to fresh sterile media (inoculum volume). During each cultivation attempt, an average of 10 tubes were inoculated per generation. This process was repeated across multiple cultivation attempts (blue), leading to variations in the total number of tubes (red) inoculated per generation. (**B**) Phosphate incubation experiment. Phosphate uptake experiment was performed on the fifth generation of P-starved cultures. During each incubation, samples were taken before phosphate addition to the mat (time 0) and after 10 min, 30 min, 2 h, and 24 h of incubation. The experiment was repeated five times using a new set of cultivation tubes. This resulted in 30 culture tubes being sacrificed for each of the five separate incubation experiments.

Each generation was cultivated under controlled conditions at room temperature (20–21°C) in the dark for 6 days. To create a new generation, a sub-sample (250–330 µL) of the bacterial mat was harvested from the previous generation using a sterile Pasteur glass pipette and transferred to freshly prepared medium without an additional P source approximately 1 cm below the air-agar surface interface (Fig. 1A). During each cultivation attempt, which took place on different days, a set of approximately 40 tubes was prepared, and an average of 10 tubes were inoculated per generation. Specifically, we performed 15 cultivation attempts for the first generation, 14 for the second generation, 15 for the third generation, 10 for the fourth, and 6 for the fifth. This resulted in a total of 106, 155, 180, 113, and 79 tubes inoculated for the first through fifth generations, respectively.

The formation of a distinct white mat 2–3 days after inoculation was an indication of successful growth. The mat establishment rate for each generation was calculated as the ratio of the number of tubes where a clear bacterial mat was established after 6 days of growth to the total number of tubes inoculated for a specific generation during each individual cultivation attempt.

### Phosphate incubation experiment

A phosphate incubation experiment was performed on the fifth P-starved generation, after 6 days of growth; 100 µL of 0.5 mmol L^-1^ KH_2_PO_4_ dissolved in artificial seawater was added directly into the mat by injecting the phosphate solution using a pipette tip, which was randomly inserted multiple times across the bacterial mat (Fig. 1B). Throughout the incubation period, samples were collected immediately after adding P-supplement (within the first 10 min, see explanation below) and after 30 min, 2 h, and 24 h of incubation. Additionally, we collected P-starved mats (time 0), blank samples with sterile medium without P-supplement, and blanks of sterile medium after P addition. Because sampling for dissolved fractions was performed using rhizons, it was technically challenging to obtain samples at precise time points. On average, it took 10 min to obtain a sufficient sample volume for further analysis. Therefore, this time interval was used as a reference point for analyzing the data of samples collected immediately after the addition of phosphate to the bacterial mat. Consequently, all other time points were shifted by this 10 min time interval to maintain consistency.

At each sampling time point, entire bacterial mats from six culture tubes were harvested (1.2 mL each) using a Pasteur glass pipette and combined into a pooled sample in a Falcon tube, which was then homogenized by repeated pipetting up and down (Fig. 1B). To slow down metabolic activity, culture tubes and pooled samples were chilled in ice during sample processing. The pooled and homogenized sample was then subsampled for the following parameters: total P, protein content, total dissolved P, dissolved inorganic phosphate, and filaments for microscopy. For the dissolved fractions, samples were collected using rhizons (Rhizosphere Research Products, Wageningen, The Netherlands). The entire experiment was repeated five times with five parallel sets of tubes over a period of 1 month to acquire five independent iterations.

As a follow-up experiment, we additionally performed short-term incubation to assess how quickly incorporated phosphate appears in the polyP pool by microscopy. Filaments were collected from the same tube at intervals of 2 min over a 30-min incubation period upon phosphate addition to the starved bacterial mat (fifth generation). The experiment was performed on three different tubes.

### Laboratory measurements

Total and total dissolved P were measured by Inductively Coupled Plasma Optical Emission Spectroscopy (ICP-OES) at 177.495 nm wavelength with external matrix-matched calibration and online addition of Y as an internal standard. Although the total dissolved P fraction was directly measured with ICP-OES after acidification with nitric acid to 2 vol%, the sample material for total P determination was first dried and then digested in closed Teflon vessels at 180°C for 12 h with a mixture of concentrated nitric (2 mL) and perchloric (1 mL) acid. After evaporation of the acids, the digestions were fumed off three times with 1 mL 6 M HCl and finally dissolved in 1.5 mL 2 vol% nitric acid. Precision and trueness of the measurement were checked by using the international reference materials SLRS-6 and CASS-6 (NRC) spiked with P and were better than 2.7% and 3.4%, respectively. The total particulate P fraction was calculated by the difference between the total and total dissolved P.

Protein content and dissolved inorganic phosphate were measured using a SPEKOL 1500 UV VIS Spectrophotometer (Analytik Jena AG, Jena, Germany). Dissolved inorganic phosphate was measured using a standard colorimetric method with ascorbic acid (37).

Protein content was determined by the Bradford method (38) using Bovine Serum Albumin Protein Standard II (Bio-Rad Laboratories, Inc., Hercules, CA, USA). Prior to measurement, the protein was extracted from 1 mL of the sample with 10% TCA (vol/vol) as described previously (39) with slight modifications: after acidic hydrolysis of the agar at 90°C at 500 rpm, a cooling step was performed on ice for 40 min, and the final pellet was dissolved at 55°C for 30 min at 250 rpm. Sterile top agar was used as a blank sample.

Polyphosphates were visualized by staining filaments with 10 mg L^−1^ 4′,6-Diamidino-2-phenylindole dihydrochloride (DAPI) solution as described previously (40). Before staining, 0.5 mL of the sample was fixed with 4% formaldehyde (vol/vol) and stored at 4°C for up to 24 h. The polyP inclusions were visualized using a fluorescence microscope (Axioskop 2 Mot PLUS, Carl Zeiss, Jena, Germany) with 360/40 nm excitation wavelength. The DAPI-polyP complex exhibits a maximum fluorescence emission of 525–550 nm, which results in polyP visualization with a bright yellow-green signal (41). With this method, polyP of at least 15 Pi residuals is visualized on microscopic images (42).

To determine the proportion of *Beggiatoa* filaments containing polyP, we counted filaments containing polyP, regardless of the number and size of granules, and calculated the ratio to the total number of filaments observed on the microscopic slide.

### Data analysis

Visualization and statistical data analysis were implemented in R (version 4.3.2) (43). To compare the median of mat establishment rates across generations, the non-parametric Kruskal-Wallis test was used, followed by post-hoc Dunn’s test for multiple comparisons using the Benjamini-Hochberg (BH) method using the FSA package (version 0.9.6) (44).

Phosphate uptake rates were calculated assuming a linear behavior between two time points for each iteration of the experiment independently, based on which the mean and standard deviation values for each time period were derived. The uptake rate per protein was calculated based on the mean value of the protein content for a specific iteration. To convert the uptake rate per protein to uptake per biovolume of the filaments, an average conversion coefficient of 39 (45) was used.

Furthermore, an exponential decay model was employed to describe the phosphate concentration over time, represented by the equation: ${Conc}_{PO_{4}^{3-}}=intercept+\alpha \cdot {exp}^{-k\cdot time}$. The model was fitted to the data using non-linear least squares regression via the nls() function, with initial parameter estimates set to α = 30, k = 0.5, and intercept = 1. An asymptotic regression was fitted to the particulate P concentrations over time with the drm() function from the drc package (version 3.0.1) (46), employing the self-starter function DRC.asymReg() from the aomisc package (47).

The data are accessible in the following repository (48).

## RESULTS

**Fig 2:**
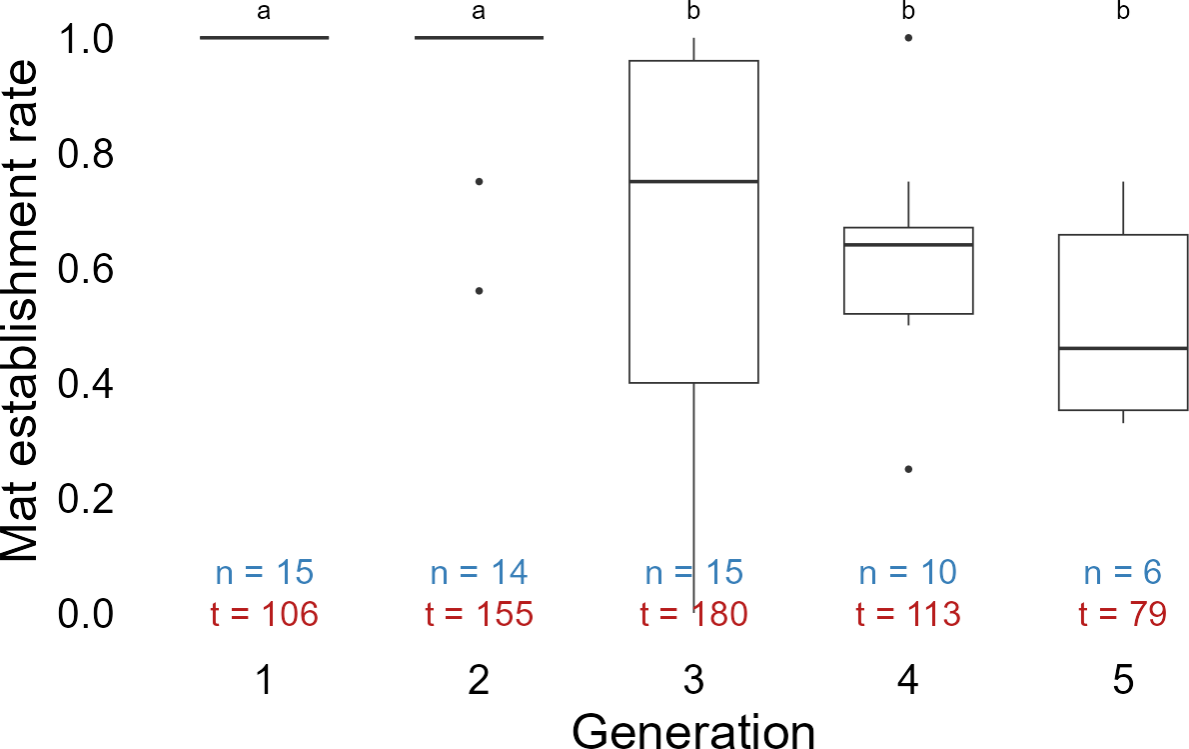
Mat establishment rate of P-starved *Beggiatoa* sp. 35Flor cultures over five generations. Boxes show the first and third quartiles; horizontal line: median; whiskers: 1.5 times the interquartile range from the edges of the box; dots represent outliers. n indicates the number of independent cultivation attempts, with each involving an average of 10 inoculated tubes. t is the total number of tubes inoculated per generation during the experiment. Letters (a and b) indicate significant differences (Dunn’s test, BH-adjusted *P*-value < 0.05).

A clear drop in mat establishment rate occurred in the third generation when the median value significantly decreased to 0.75 compared with the first and second generations (Fig. 2). By the fifth generation, which was used for the main phosphate incubation experiments, the bacterial mat establishment rate further decreased to 0.46. No significant change was detected in mat establishment rate between generations three and five.

**Fig 3:**
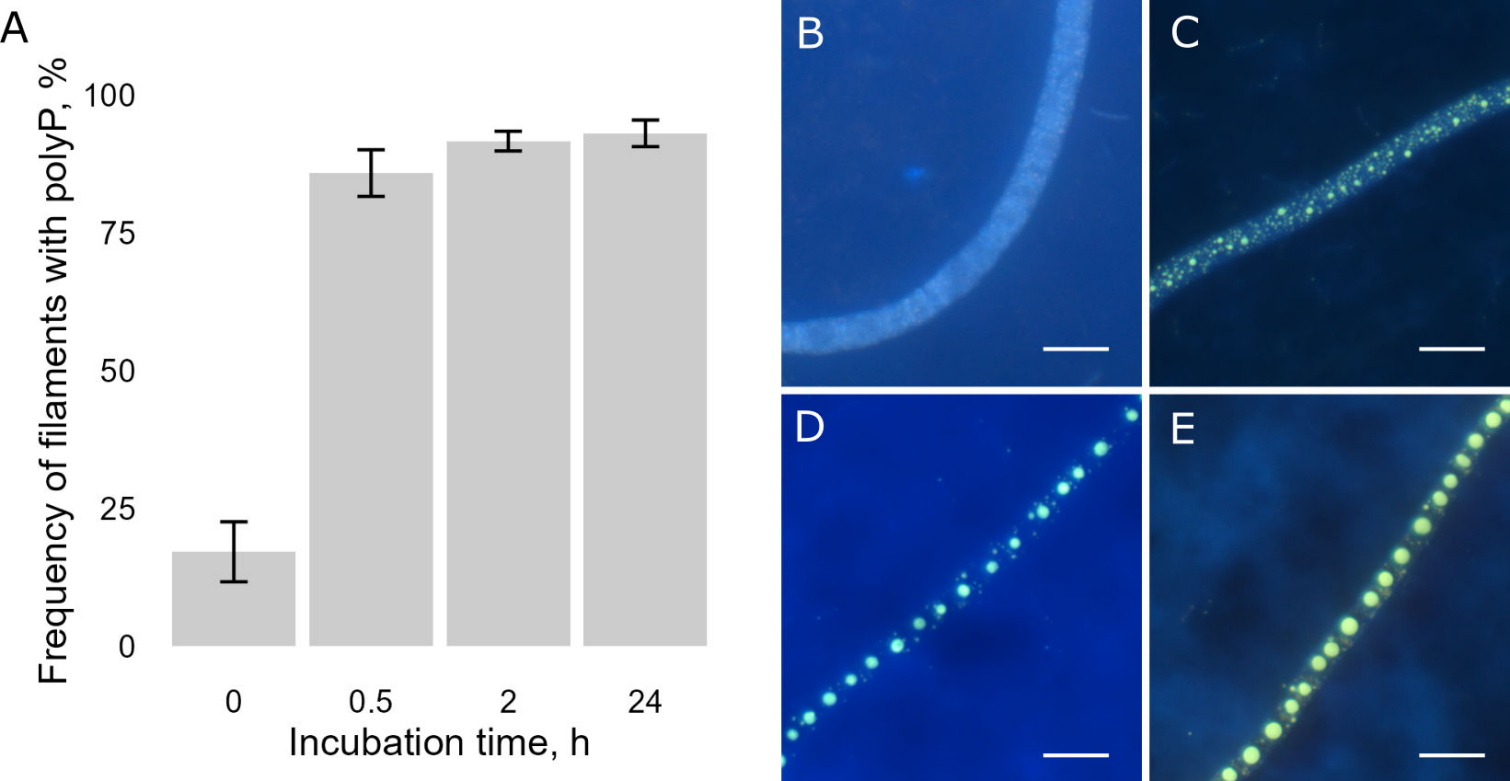
Polyphosphate dynamics in *Beggiatoa* sp. 35Flor following the addition of phosphate to the P-starved cultures. (A) Percentage of filaments containing polyphosphates (indicated as yes/no) over the course of the experiment; error bars show the standard deviation (*n* = 5). (B–E) DAPI-stained fluorescence images of *Beggiatoa* sp. 35Flor cultures at different times of incubation: (B) P-starved culture at the beginning without P-addition (time 0), and (C) after 30 min, (D) after 2 h, and (E) after 24 h of incubation with P. Bars represent 10 µm.

Fluorescence microscopy of DAPI-stained samples derived from cultures subjected to phosphate starvation for five generations showed that 83% of the filaments did not have any detectable polyP inclusions prior to phosphate addition. However, within 30 min upon P-addition, the number of filaments containing at least some polyP increased to 86% and reached 93% by the end of the 24 h of incubation (Fig. 3A). After 30 min of incubation, a clear yellow signal of small round granules was visible, which became larger, emitted a more intense signal after 2 h, and further intensified after 24 h of incubation. The polyP distribution varied across different filaments and within individual filaments. The DAPI-stained filaments (Fig. 3B through E) showed a shift from numerous small granules after 30 min incubation toward storing polyP in the center of the cell within the main vacuole, reaching diameters of up to 5 µm by the end of incubation after 24 h.

The average background phosphate concentration in the sterile cultivation media was 0.2 µmol L^−1^. At the beginning of the phosphate incubation experiment, when the bacterial mat was established in the fifth P-starved generation, phosphate concentration decreased to below the detection limit (0.10 µmol L^−1^) for three iterations, and for the other two, it was measured at 0.11 and 0.12 µmol L^−1^. At the start of the experiment, the addition of KH₂PO₄ to the media resulted in an average initial phosphate concentration of 39 µmol L⁻¹ measured in sterile media. Extremely high uptake rates (Table 1) were detected for the time interval between 0 and 10 min, followed by a gradual decrease throughout the rest of the incubation period.

**TABLE 1:** Mean phosphate uptake rates of *Beggiatoa* sp. 35Flor at different time intervals assuming a linear relationship^*a*^

Time interval	Uptake rate per g protein, mmol g^−1^ d^−1^	Uptake rate per biovolume, mmol L^−1^ d^−1^
0–10 min	298 ± 98	11,636 ± 3,857
10–30 min	132 ± 56	5,170 ± 2,191
30 min to 2 h	31 ± 7	1,202 ± 294
2–24 h	3.2 ± 0.6	124 ± 23

^*a*^ The values are given as mean ± standard deviation of five iterations of the experiment.

**Fig 4:**
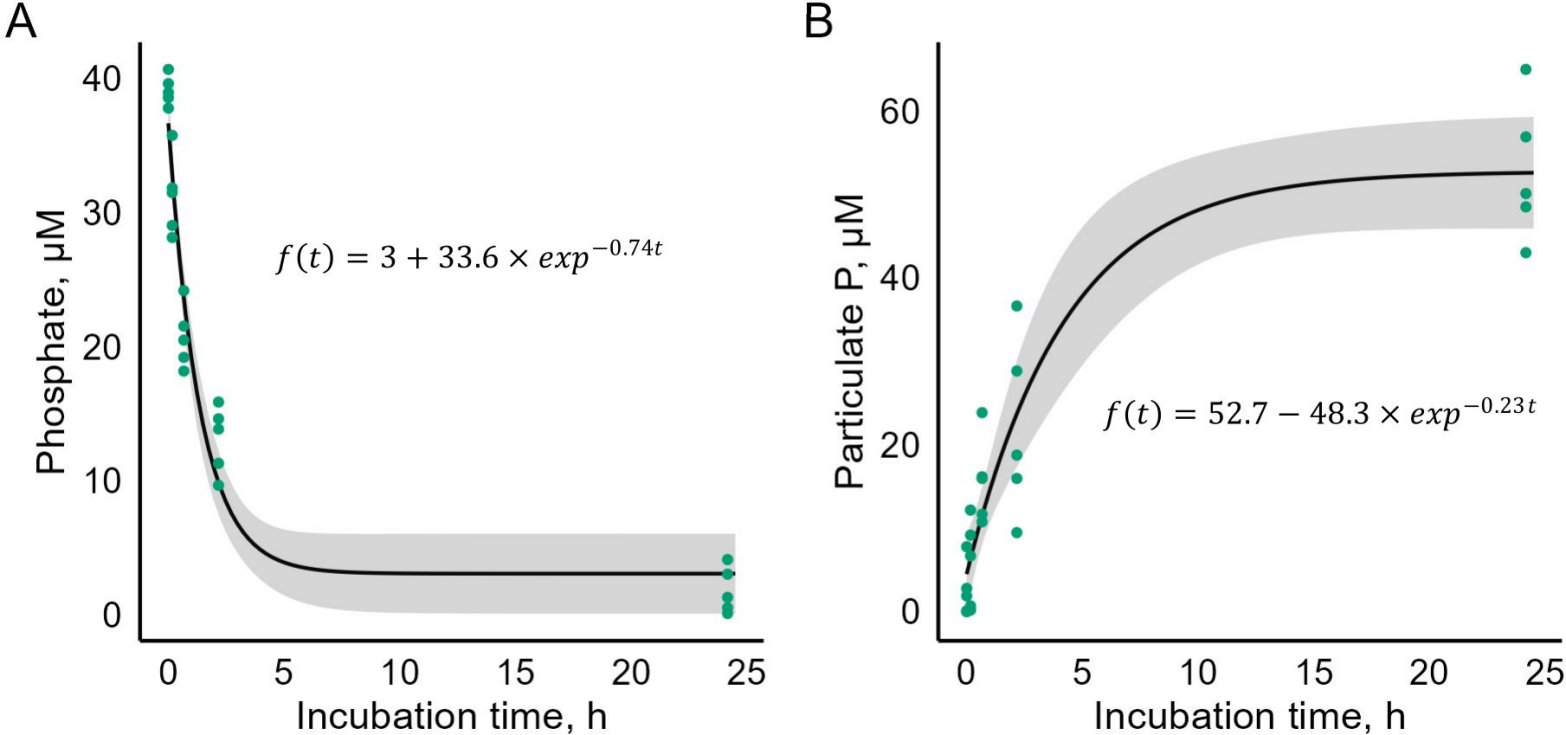
Concentrations of dissolved inorganic phosphate (**A**) and particulate P (**B**) over incubation time. Each dot represents a measurement from each of the five iterations of the experiment. The black line shows the fitted curve, and the gray ribbon shows the 95% CI for the fitted curve.

The phosphate concentration decline over time (Fig. 4A) is well described by an exponential decay model (*P*-value < 0.05 for all coefficients). A nearly 2-fold reduction was observed within the first 30 min, and the concentration continued to decrease, reaching an average of 1.78 µmol L⁻¹ after 24 h of incubation.

The opposite trend was observed for particulate P (Fig. 4B), which matched the asymptotic regression (*P*-value < 0.05 for all coefficients). Rapid accumulation of particulate P occurred within the first 30 min, and the bacterial mat continued to accumulate P until the final sampling point, after 24 h of incubation.

## DISCUSSION

### Mat establishment rate at different generations of P-depleted cultures

Healthy *Beggiatoa* spp. populations, in nature and under culture conditions, aggregate in mats located at the oxic-anoxic interface to obtain access to sulfide and oxygen, the redox couple that they use for energy generation (25, 36). This behavior is driven by negative oxygen taxis and was observed even in the absence of sulfide (49). In this study, we observed a significant drop in the mat establishment rate of *Beggiatoa* sp. after three generations of P-starved cultures (Fig. 2). Although bacteria have a very low cell quota for P in general (50), phosphorus starvation nevertheless induces various changes, including, but not limited to, reduced growth and poor survival (51, 52), which align well with our findings. However, it is possible that P starvation indirectly influenced the ability to establish bacterial mat in *Beggiatoa* sp. 35Flor culture.

During the current experiment, tubes without an established bacterial mat, in most cases, still contained motile individual filaments, that were distributed across a broad depth range, suggesting their presence in both the sulfidic and the oxic layers. However, these filaments failed to aggregate at the oxygen-sulfide interface and did not show an increase in biomass in the later phases for reasons that are not fully understood. A similar distribution pattern of filaments was observed under conditions when the sulfide and oxygen zones did not overlap (53). This caused filaments to glide longer distances while using internally stored nitrate as an alternative electron acceptor. It is possible that the urgent need for P overrides the typical chemotactic response of filaments to oxygen, causing them to glide beyond the oxygen-sulfide interface to scavenge for available phosphate. As a result, cells may die because of the inability to generate sufficient energy from sulfide oxidation caused by a mismatch of electron donors and acceptors. At the same time, gliding of filaments into oxygenated parts of the media may lead to cell death due to their inability to mitigate oxidative stress, as *Beggiatoa* spp. typically lack the catalase enzyme critical for the oxidative stress response (54).

An alternative explanation for the inability to form a mat could be attributed to the reduced gliding speed resulting from the missing polyP as an energy source. A link between polyP and swimming motility was postulated for pathogenic bacteria (55), where the authors observed reduced motility due to the absence of polyP in mutant strains. A similar connection has also been hypothesized for the polyP-accumulating chemolithoautotrophic sulfur bacteria *Sulfurimonas* subgroup GD17, inhabiting the pelagic redox zone of the Baltic Sea (56). The authors linked the energy released from the degradation of polyP to the energy required for the movement of the flagella. Consequently, polyP or P starvation in the present study can reduce the ability to find the optimal position across the oxygen-sulfide gradient in the media to sustain growth.

### Crucial role of polyP in survival

We decided to perform the phosphate incubation experiment on the fifth generation of P-starved cultures because only this generation demonstrated a reduction in polyP in the majority (83%) of the filaments (Fig. 3A, time 0), as evidenced by the absence of the yellow fluorescence signal following DAPI staining. During starvation, no additional P was added to the cultures; only traces of P from different media components (mostly agar) were available (mean 0.2 µmol L^−1^) for the bacteria. Besides that, conservative estimations show that through the dilution of the original stock culture (180 µmol L^−1^ phosphate concentration), only 0.12 µmol L^−1^ could be carried over to the fifth generation (see Text S1 in Supplemental Material). Considering such sustained P-depleted conditions, it was surprising that *Beggiatoa* sp. 35Flor filaments were able to grow for at least five generations, indicating their remarkable resilience to different phosphate levels.

Even in the fifth generation of P-starved cultures, approximately 17% of the *Beggiatoa* sp. 35Flor population still contained visible polyP inclusions (Fig. 3A, time 0). The presence of polyP inclusions even at such sustained P-depleted conditions could indicate that polyP not only acts as a reservoir of inorganic phosphate for other molecules (for example, ATP and phospholipids) but may also play an important role in more specific metabolic processes related to critical survival mechanisms. Previous research demonstrated that under various stress conditions, polyP is linked to a general stress response by inducing the expression of rpoS genes responsible for transcription-regulating sigma factors (10). The link between polyP and stress response was confirmed for *Beggiatoa* sp. 35Flor strain by Langer et al. (57). The authors showed that the polyP turnover rate in this strain remains high under both favorable (oxic, low sulfide flux) and stressful conditions (anoxic, high sulfide flux), underlining the importance of polyP in stress response and subsequent survival metabolism. Based on the decline in the mat establishment rate across generations, we can assume that long-term P starvation, to which *Beggiatoa* sp. 35Flor was exposed to in our study, caused stressful growth conditions stimulating a stress response. Furthermore, we hypothesized that the absence of polyP due to starvation could potentially suppress metabolic survival mechanisms and reduce the chances of successful mat establishment.

### Overplus rapid phosphate uptake and polyP formation

After adding phosphate to the starved cultures, we observed extremely high phosphate uptake rates by the marine sulfur bacterium *Beggiatoa* sp. 35Flor. In general, nutrient uptake in bacteria can be mathematically described by the Michaelis-Menten kinetic equation, which considers enzyme-catalyzed reactions of active transport. This model assumes the rates to be dependent on the initial substrate concentration and is limited by the number of enzymes present in the cell membrane (58). As such, the theoretical maximum overplus phosphate uptake rate for *Acinetobacter* spp., typical bacteria of activated sludge from wastewater treatment plants, has been estimated at 29 mg P g⁻¹ dry mass (DM) h⁻¹, derived from initial uptake measurements within 15–30 min after phosphate addition (14). This is equivalent to 1.5 mmol phosphate g^−1^ protein h^−1^, based on the DM-to-protein conversion factor of 0.63 (59). For *Beggiatoa* sp. 35Flor mats, we calculated an initial phosphate uptake rate of 12.4 mmol g^−1^ protein h^−1^ over the first 10 min of incubation. This rate is more than eight times higher than reported for *Acinetobacter* spp. This discrepancy could be attributed to the larger cell volume of *Beggiatoa*, which allows greater polyP storage. A reasonable comparison for *Beggiatoa* sp. 35Flor would be with filamentous bacteria of similar sizes, such as freshwater cyanobacteria *Oscillatoria agardhii*, which has been shown to form polyP directly after overplus phosphate uptake (60). The phosphate uptake rate for *O. agardhii* under P-depleted conditions was up to 3.1 mmol g^−1^ protein h^−1^, measured at 1-minute intervals after phosphate addition (61) and thus is nearly four times lower than for *Beggiatoa* sp. 35Flor. Given the comparable sizes of their filaments, other factors may explain the significant differences in uptake rates. As such, these variations could be attributed to the duration of P starvation, differences in the considered low P concentrations in the experimental design or could lay in the differences in physiology and the genetic make-up of different bacterial species.

The rapid uptake of phosphate observed during the initial 10 min of incubation agrees with the typical overplus response observed in starved bacteria. In phosphate-deprived environments, most bacteria induce the high-affinity phosphate transport system PstSCAB, which actively transports phosphate (62). After phosphate is reintroduced, microorganisms switch from the high-affinity PstSCAB to the low-affinity PitH phosphate transport systems (62). This switch is not instantaneous, leading to fast initial uptake rates. Such a phenomenon has been well-documented in cyanobacteria (63, 64), various heterotrophic bacteria (65–67), and yeast (68). Because our first sample was collected at a maximum of 10 min after phosphate addition, the potential effect of the high-affinity transport system may have been underestimated, as previous studies have shown that this process can occur within a few minutes (63). This hypothesis is also supported by our observations of polyP inclusions formed during short-term incubation. Notably, the polyP began to appear after only 2 minutes of incubation with phosphate (see Fig. S1 in Supplemental Material), which is consistent with observations made for *Nostoc* sp. by Solovchenko et al. (64). The authors postulated that such rapid polyP transport into vacuoles can serve as a safety mechanism to prevent the accumulation of short-chain polyP due to its potential toxicity coming from scavenging divalent cations. Indeed, this effect has been documented in yeast cultures, and the incorporation of polyP into acidocalcisomes was proposed as a protective mechanism (69). This assumption is consistent with the observation for *Beggiatoa* sp. 35Flor, where polyP was demonstrated to be stored in acidocalcisome-like vacuoles (70).

### Potential effect of *Beggiatoa* spp. mats on P cycling

We could not directly measure polyP due to challenges in extracting polyP from the bacteria-agar mixture and therefore cannot conclusively state that all the assimilated phosphate was converted into polyP. Nevertheless, our microscopic observations (Fig. 3B through E) strongly support the hypothesis of phosphate storage in the form of polyP in *Beggiatoa* sp. 35Flor. We calculated that 100% of the added phosphate was incorporated into the particulate pool within 24 h of incubation (see Text S2 in Supplemental Material). Since we did not detect a notable increase in the protein content of the samples, we could therefore assume that at least a large proportion of the consumed phosphate was stored as polyP, although other intracellular P pools, such as RNA and lipids (71), could not be excluded. Although the accompanied bacterium *Pseudovibrio* sp. strain FO-BEG1, which is present in low numbers in the *Beggiatoa* culture, can also form two polyP inclusions up to 0.5 µm in diameter, their contribution to polyP inclusion volume is at least two orders of magnitude smaller than that of *Beggiatoa* sp. 35Flor (see Text S3 in Supplemental Material) and thus is negligible.

To estimate the possible effect of *Beggiatoa* spp. mats on fluctuating phosphate concentrations under environmental conditions, we used the phosphate uptake rate determined after 30 min of incubation, the 30 min to 2 h and 2–24 h periods, which may be considered luxury uptake. This is supported by the observation that the filaments already contained polyP granules (Fig. 3B through E) to the extent similar to the growing conditions with P excess in the initial stock culture and are thus not substantially phosphate-deprived. This phosphate luxury uptake ranges between 124 (2–24 h) and 1,202 (30 min – 2 h) mmol L^−1^ d^−1^ (Table 1). To extrapolate the obtained phosphate uptake to *in situ* conditions, the density of filaments in natural habitats should be considered. The biovolume of *Beggiatoa* spp. filaments has been estimated in only a few published studies, as summarized in Table 2. These studies cover a range of environments, including intertidal mud flats and the brackish sediments of Limfjorden, which share characteristics with other coastal and brackish ecosystems. However, these locations were selected based on detectable *Beggiatoa* spp. biomass and are not representative of a randomized survey of potential habitats. Therefore, the extrapolated phosphate uptake rates should be interpreted as estimates for environments where *Beggiatoa* spp. populations are well documented, rather than a global assessment of their potential uptake rates. Assuming conservatively an average *Beggiatoa* spp. biovolume of 5 cm^3^ m^−2^ across the locations listed in Table 2 and applying the range of phosphate uptake rates per biovolume of filaments calculated in this study (124–1202 mmol L^−1^ d^−1^), the average phosphate uptake of such natural environments would be approximately 0.6–6 mmol m^−2^ d^−1^. These values are of the same order of magnitude as the phosphate uptake rate of 1.2 mmol m^−2^ d^−1^ detected in Namibian sediments under oxic conditions, where the activity of large sulfur bacteria was studied using a ³³P-radiotracer in laboratory incubations (21). The authors linked bacterial phosphate uptake to apatite formation and postulated the importance of sulfide-oxidizing bacteria as P sinks. Similarly, an earlier study from the same location linked phosphorite deposits to the activity of *Thiomargarita namibiensis*, indicating that large sulfur bacteria are crucial in driving phosphorite formation under anoxic conditions (20).

**TABLE 2:** Overview of *Beggiatoa* spp. distribution, biovolume, and filament diameter across different locations integrated over 5 cm sediment depth

Location	Environment	Biovolume, cm^3^ m^−2^	Filament diameter range (dominant), µm	Reference
Limfjorden, Denmark	Brackish sediment, 4–12 m water depth	5–20	3–35 (15)	(24)
Limfjorden, Denmark	Brackish sediment, 4–12 m water depth	14–16	3–40	(25)
Wadden Sea (Dangast/Jadebay)	intertidal mud ﬂat	0.6–1	9–12	(25)
Svalbard	Fjord sediments	1.13–3.36	2–50 (2–5)	(26)

So far, *Beggiatoa* spp. and other large sulfur bacteria have been primarily associated with phosphate release under anoxic and highly sulfidic conditions (22, 23, 72). However, less attention has been paid to their functional roles related to the P cycle in ecosystems where the formation of P-associated minerals does not occur. *Beggiatoa* mats have been reported from various locations, including the shallow coastal waters of Denmark and Germany (24, 25), intertidal flats (25, 32), coastal zones of Chile (73), mangrove sediments (74), Arctic Ocean (26, 33), deep sea (29, 31), as well as freshwater habitats (27) and hypersaline lakes (30). However, *Beggiatoa* spp. do not always form consistently noticeable white mats at the sediment surface and could thus be easily overlooked. Detailed sediment surveys have revealed that typically the peak of *Beggiatoa* spp. biomass occurs below the sediment surface (24, 25), potentially leading to an underestimation of these groups on a larger spatial scale.

Assuming similar *Beggiatoa* spp. biovolumes as those reported in Table 2, we hypothesize that the phosphate uptake rate and polyP storage capacity of *Beggiatoa* spp. are sufficient to quantitatively affect phosphate release into bottom waters coming from diffusive porewater fluxes originating in deeper sediments. We propose that under oxygenated conditions with low sulfide fluxes, *Beggiatoa* spp. could function as a temporary P sink in sediments owing to their high phosphate accumulation capacity. However, when exposed to sulfidic conditions and anoxia, *Beggiatoa* spp. break down polyP and release previously stored phosphate in large quantities substantial enough to contribute to the total phosphate flux from the sediment into the overlaying waters (22, 72). These dynamics demonstrate the tipping point at which the polyP pool associated with *Beggiatoa* mats shifts from a P sink to a P source is determined by their sensitivity to redox conditions. The change from oxic to anoxic conditions is typically subjected to temporal variability and in some cases corresponds with seasonal fluctuations, as was shown, for example, for the Baltic Sea sediments (72).

In conclusion, our study demonstrates that *Beggiatoa* sp. strain 35Flor can tolerate prolonged P depletion, likely facilitated by polyP storage, which appears to play a crucial role in bacterial mat establishment under P-starved conditions. We also observed exceptionally rapid overplus phosphate uptake rates under P reintroduction to starved culture, which highlights the unique physiological capabilities of this bacterium. Although further studies involving environmental samples are necessary to confirm the role of *Beggiatoa* spp. mats in moderating sediment-water interface phosphate fluxes, our findings suggest that *Beggiatoa* mats have the potential to act as a significant P sink in aquatic environments under oxic conditions.

## SUPPLEMENTAL MATERIAL

Supplemental material aem.02330-24-s0001.pdf Text S1 to S3; Fig. S1.

ASM does not own the copyrights to Supplemental Material that may be linked to, or accessed through, an article. The authors have granted ASM a non-exclusive, world-wide license to publish the Supplemental Material files. Please contact the corresponding author directly for reuse.
