# Supplementary material for: Distinctive physiology of polyphosphate-accumulating Beggiatoa suggests an important role in benthic phosphorus cycling
Source: Appl Environ Microbiol. 2025 Apr 30;91(5):e02330-24. doi: 10.1128/aem.02330-24 (PMC12094007; doi:10.1128/aem.02330-24)
Supplement: Supplemental material — Text S1 to S3; Fig. S1. [file aem.02330-24-s0001.pdf]

Supplemental Material for

Distinctive physiology of polyphosphate-accumulating *Beggiatoa* suggests an important role in benthic phosphorus cycling

Authors: Nadezhda Iakovchuk[a,b], Jenny Fabian[a], Olaf Dellwig[c], Christiane Hassenrück[a], Heide N. Schulz-Vogt[a,b]

[a]Department of Biological Oceanography, Leibniz Institute for Baltic Sea Research Warnemünde, Rostock, Germany

[b]Faculty of Mathematics and Natural Sciences, University of Rostock, Rostock, Germany

[c]Department of Marine Geology, Leibniz Institute for Baltic Sea Research Warnemünde, Rostock, Germany

#Address correspondence to: Nadezhda Iakovchuk, nadezhda.iakovchuk@io-warnemuende.de

Text S1. Phosphate carried over from high concentration phosphate stock culture through generations

The initial stock culture had phosphate concentration of approximately 180 µmol L^{-1}. During transfers across generations, 250 µL of culture was inoculated for generations 1–3, and 330 µL for generations 4–5. Assuming the inoculated media with filaments was diluted only in the sampled volume of 1.2 mL, the estimated phosphate concentrations carried over through five generations are:

1st generation: (180 µmol L^{-1} × 250 µL)/1.2 mL = **37.5** µmol L^{-1}

2nd generation: (37.5 µmol L^{-1} × 250 µL)/1.2 mL = **7.8125** µmol L^{-1}

3rd generation: (7.8125 µmol L^{-1} × 250 µL)/1.2 mL = **1.6276** µmol L^{-1}

4th generation: (1.628 µmol L^{-1} × 330 µL)/1.2 mL = **0.4476** µmol L^{-1}

5th generation: (0.4476 µmol L^{-1} × 330 µL)/1.2 mL = **0.1231** µmol L^{-1}

This calculation assumes that the phosphate remained within the sampled 1.2 mL volume. However, this is a conservative estimate, as it does not account for phosphate diffusion beyond the sampled volume, which would further dilute the carried-over phosphate concentration. Moreover, since the original stock culture was also maintained in a gradient medium, the phosphate concentration at the site of the *Beggiatoa* mat may have been lower due to phosphate uptake for growth and polyphosphate storage, which further reduced the phosphate concentration.

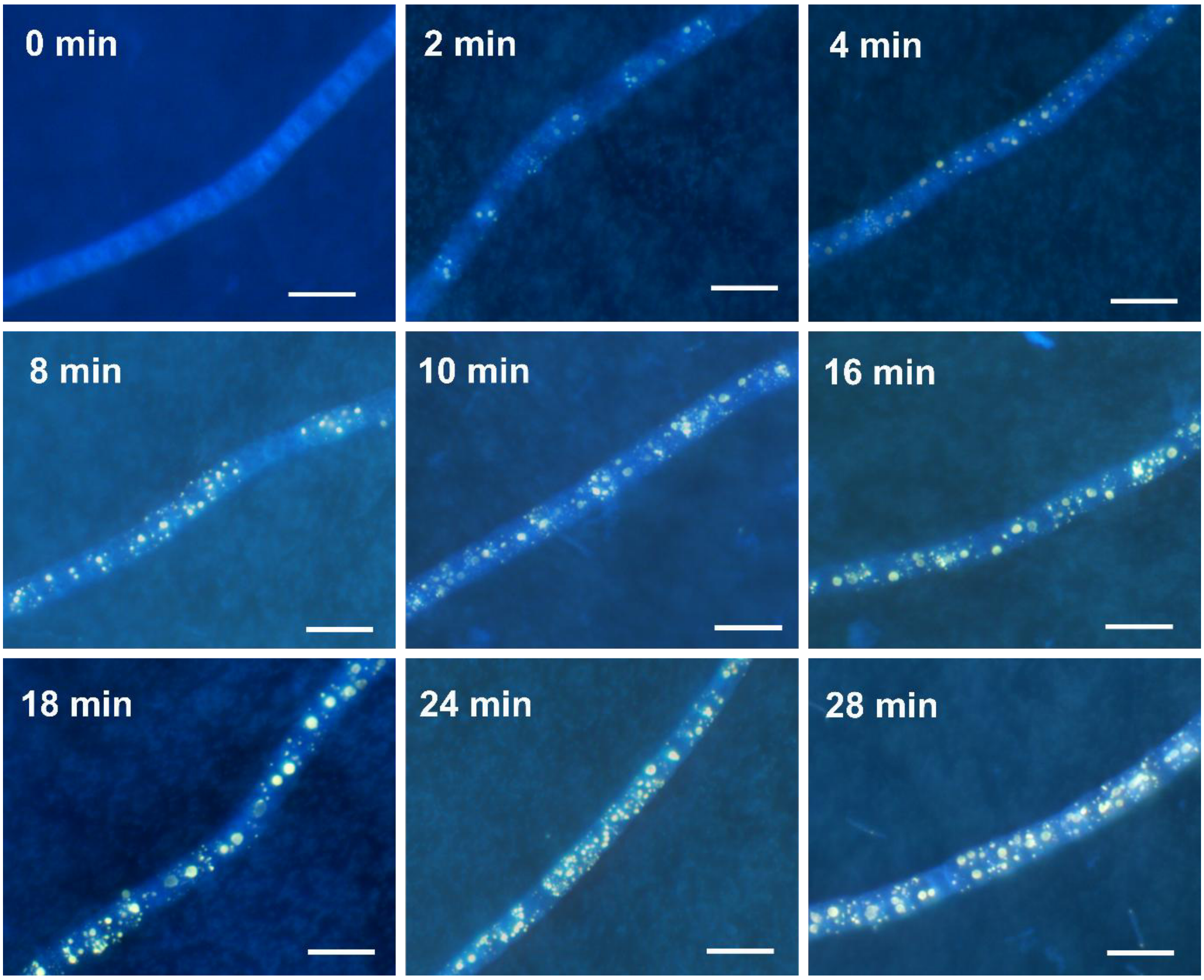

Figure S1. DAPI-stained fluorescent images of *Beggiatoa* sp. 35Flor cultures from short-term phosphate incubation experiment with 2 min sampling intervals. 0 min – P starved filaments before addition of phosphate. Scale bar is 10 µm.

Text S2. Proportion of added phosphate being transferred into cellular polyP

Assuming the theoretical maximum of P amount (in µmoles) in the phosphate incubation experiment as the sum of supplied P in the form of phosphate of 0.05 µmol P (when calculated from 100 µL × 0.5 mmol stock solution added to the mat) and the initial P content of the starved bacterial mat of 0.006 µmol P (5.0 µmol L^{-1} × 1.2 mL sample taken), the available P during the incubation would be **0.056 µmol**. At the 24 h incubation time point, the mean particulate P was 52.7 µmol L^{-1}, which is equivalent to **0.0632 µmol** of P (52.7 µmol L^{-1} × 1.2 mL sample taken). This value is slightly higher (about 12%) than the assumed theoretical maximum of P in the incubation. However, considering the analytical error of the measurement, this would indicate 100% incorporation of added P into the particulate pool.

Text S3. Volume ratio of polyphosphate inclusion in *Beggiatoa* sp. 35Flor and *Pseudovibrio* strain FO-BEG1

In Fig. 3.E, a part of the *Beggiatoa* sp. 35Flor filament is depicted together with several *Pseudovibrio* FO-BEG1 cells. We counted 18 individual *Beggiatoa* sp. 35Flor cells, identified by their central vacuoles filled with polyP, typically ranging from **3 to 5 µm** in diameter. Additionally, three to five *Pseudovibrio* FO-BEG1 cells are commonly observed in similar microscopic images. Typically, *Pseudovibrio* FO-BEG1 forms two polyP inclusions on opposite poles of the cell with diameter **below 0.5 µm**. To conservatively compare the potential polyP volumes in a typical sample, we considered 10 single *Beggiatoa* sp. 35Flor cells with 3 µm polyP inclusions and 5 *Pseudovibrio* FO-BEG1 cells with 0.5 µm polyP inclusions. This very conservative estimation reveals a volume difference of two orders of magnitude in favor of *Beggiatoa* sp. 35Flor.
